# NMDA receptor activation inhibits the antifibrotic effect of BM-MSCs on bleomycin-induced pulmonary fibrosis

**DOI:** 10.1152/ajplung.00002.2018

**Published:** 2018-05-03

**Authors:** Xiaohong Li, Chen Li, Yiting Tang, Yanhong Huang, Qingmei Cheng, Xiaoting Huang, Feiyan Zhao, Caixia Hao, Dandan Feng, Jianping Xu, Jianzhong Han, Siyuan Tang, Wei Liu, Shaojie Yue, Ziqiang Luo

**Affiliations:** ^1^Department of Physiology, Xiangya School of Medicine, Central South University, Changsha, Hunan, China; ^2^Department of Pediatrics, Xiangya Hospital, Central South University, Changsha, Hunan, China; ^3^Department of Physiology, Changzhi Medical College, Changzhi, Shanxi, China; ^4^Xiangya Nursing School, Central South University, Changsha, Hunan, China

**Keywords:** bleomycin-induced pulmonary fibrosis, bone marrow-derived mesenchymal stem cell, glutamate, *N*-methyl-d-aspartate receptor

## Abstract

Endogenous glutamate (Glu) release and *N*-methyl-d-aspartate (NMDA) receptor (NMDAR) activation are associated with lung injury in different animal models. However, the underlying mechanism is unclear. Bone marrow-derived mesenchymal stem cells (BM-MSCs), which show potential use for immunomodulation and tissue protection, play a protective role in pulmonary fibrosis (PF) process. Here, we found the increased Glu release from the BM cells of bleomycin (BLM)-induced PF mice in vivo. BLM stimulation also increased the extracellular Glu in BM-MSCs via the antiporter system x_c_^−^ in vitro. The gene expression of each subunit of NMDAR was detected in BM-MSCs. NMDAR activation inhibited the proliferation, migration, and paracrine function of BM-MSCs in vitro. BM-MSCs were derived from male C57BL/6 mice, transfected with lentiviral vectors carrying the enhanced green fluorescence protein gene, pretreated with NMDA, and transplanted into the female recipient mice that were intratracheally injected with BLM to induce PF. Transplantation of NMDA-pretreated BM-MSCs significantly aggravated PF as compared with that in the normal BM-MSCs transplantation group. The sex determination gene Y chromosome and green fluorescence protein genes of BM-MSCs were detected to observe BM-MSCs homing in the fibrotic lungs. Moreover, NMDAR activation inhibited BM-MSC migration by downregulating the stromal cell-derived factor-1/C-X-C chemokine receptor type 4 signaling axis. NMDAR activation aggravated the transforming growth factor-β1-induced extracellular matrix production in alveolar epithelial cells and fibroblasts through the paracrine effects of BM-MSCs. In summary, these findings suggested that NMDAR activation-mediated Glu excitotoxicity induced by BLM in BM-MSCs abolished the therapeutic effects of normal BM-MSCs transplantation on BLM-induced PF.

## INTRODUCTION

Idiopathic pulmonary fibrosis (IPF) is a chronic, progressive, and irreversible lung-restricted disease and the most common outcome of the interstitial lung diseases (33). This disease is characterized by replacement of the normal lung tissues by fibrotic scarring, honeycombing, and abnormal proliferation of myofibroblasts. IPF has no lasting therapeutic option other than transplantation, and its underlying pathogenesis remains unclear. The hallmark lesions are the fibroblast foci, in which vigorous fibroblast replication and exuberant extracellular matrix (ECM) deposition occur (5). The pathologic degree of fibroblast foci is closely related to the prognosis of IPF. Resident fibroblasts can be activated and differentiated into myofibroblasts, which express α-smooth muscle actin (α-SMA) and promote collagen protein expression and ECM deposition in damaged lung tissues (35). Alveolar epithelial cell (AEC)-derived fibroblasts are the other components of fibroblastic foci during pulmonary fibrosis (PF). These cells facilitate epithelial-mesenchymal transition (EMT), which involves continuous decrease in epithelial markers, including E-cadherin and keratin, and continuous increase in mesenchymal markers, including N-cadherin, vimentin, and α-SMA (24). Transforming growth factor-β (TGF-β) is a key factor that promotes the differentiation of fibroblasts into myofibroblasts and induces AECs to undergo EMT during PF development (67). Although Rock et al. (50) once suggested in 2011 that EMT is not a source of fibroblasts in mouse fibrotic lungs, the recent literature still mentions that EMT is one of the sources of myofibroblasts (49).

Evidence shows that bone marrow (BM)-derived fibrocytes also play a key role in establishing fibrosis at the injured lung sites (2). However, BM can also generate mesenchymal stem cells (MSCs), which have protective effects against PF. MSCs are a fibroblast-like and multipotent stromal cell population. The most studied source of MSCs is BM, also known as bone marrow-derived mesenchymal stem cells (BM-MSCs). MSCs have the potential of clonality, self-renewal, and transdifferentiation in vitro and exhibit actions that include homing, epithelial repair, bactericidal activity, immunomodulation, and secretion of growth factors, anti-inflammatory factors, and microvesicles (61). BM-MSC therapy has positively ameliorated inflammation and regulated the remodeling of fibrotic lung in multiple animal models. In 2003, Ortiz et al. (45) first reported that BM-MSCs administration through the jugular vein immediately after bleomycin (BLM) challenge can significantly reduce PF. The antifibrotic effects of MSCs were also successively validated in other PF animal models (10, 73). Data from MSC-based clinical trials support the safety of a single infusion of human MSCs in patients with IPF (19). However, the function of bone marrow and endogenous MSCs in the PF process is unknown. Some studies have showed that PF is accompanied by hyperplasia of BM hemopoiesis, fibrocytes, leukocytosis in peripheral blood, and reduced content of BM-MSCs in the animal model of BLM-induced PF (16, 46). Busulfan was administered to mice before intratracheal injection BLM to suppress BM function, and this treatment can aggravate PF. Supplementing exogenous MSCs can also reduce PF to some extent (51).

Glutamate (Glu) is a major excitatory neurotransmitter in the mammalian central nervous system (CNS) and plays an important role in learning, memory, and development (40). However, extracellular Glu can be abundantly released under pathological conditions, thus leading to overstimulation of Glu receptors and consequently to neuronal cell injury or death known as excitotoxicity (38, 44). The *N*-methyl d-aspartate (NMDA) receptor (NMDAR) is a subtype of ionotropic Glu receptor (iGluR) family and the principal receptor in mediating Glu neurotoxicity (74). NMDAR has seven subunits, namely, NR1, NR2 (A–D), and NR3 (A, B) (63). NMDAR is also expressed in nonneuronal tissues and cells, such as islets (21), lungs (13), and alveolar macrophages (56). Said et al. (52) first reported that NMDAR activation by NMDA, a synthetic agonist that selectively activates NMDAR, provokes acute edematous lung injury and this injury was reversed by MK-801, a noncompetitive channel antagonist of NMDAR. MK801 also plays protective effects in oxidant lung injury induced by paraquat or xanthine oxidase (53) and acute lung injury induced by hyperoxia (64). Memantine, another NMDAR antagonist, attenuates BLM-induced acute lung injury (31). The release of endogenous Glu mediates the newborn rat lung damage, which is induced by hyperoxia through NMDAR activation (65). These studies strongly suggest that the release of endogenous Glu and NMDAR activation play important roles in different lung injury models. In the present study, we find that NMDAR is also present in BM-MSCs. We speculate that in BLM-induced PF, BM-MSCs are NMDAR-regulated target cells, and NMDAR activation-mediated Glu neurotoxicity on BM-MSCs is involved in the development of lung fibrosis.

## MATERIALS AND METHODS

### 

#### MSC culture.

MSCs were derived from BM of male C57BL/6 mice in this study. The primary BM-MSCs were isolated as previously described (70). The primary BM-MSCs at passage 3–6 were used in subsequent cell experiments. The primary green fluorescence protein (GFP)-labeled BM-MSCs were purchased from Cyagen Biosciences, (Guangzhou, China) and used at early passage (<3 passages) for subsequent animal experiments. These samples were cultured in a 1:1 mix of Dulbecco’s modified Eagle medium/nutrient mixture F-12 (DMEM/F-12; Hyclone) containing 10% fetal bovine serum (FBS; Cyagen Bioscience) and 1% antibiotics (streptomycin and penicillin) and 1% l-glutamine (GIBCO, Paisley, UK) and were incubated at 37°C in a humidified atmosphere of 5% CO_2_. For transduction with lentiviral vectors carrying enhanced GFP reporter gene, BM-MSCs were seeded at 5 × 10^4^ cells in a 25-cm^2^ culture flask. The following day, virus particles were added at a multiplicity of infection for 16 h. The cells were washed, and fresh medium with doxycycline (10 mg/ml) was added for 5 days to induce transgene expression. BM-MSCs were sorted using the flow-activated sorter Mo-Flo (Becton Dickinson, Oxford, UK) to enrich the GFP-expressing cells. The GFP-positive fraction was cultured for 7 or 10 days.

#### Characterization of MSCs.

MSCs were identified by flow cytometric analysis for surface markers and differentiation ability assays. The differentiation capacity of MSCs was detected by adding differentiation media into osteocytes, adipocytes, and chondrocytes and subsequently conducting Alizarin Red staining, Oil Red O staining, and Alcian Blue staining, respectively.

#### Flow cytometry.

MSCs were detected the expression of specific surface markers, including PE anti-mouse Sca-1, PE anti-mouse CD90.2, PE anti-mouse CD29, PE anti-mouse CD44, PE anti-mouse CD45, APC anti-mouse CD31, APC anti-mouse CD34, and APC anti-mouse CD117 (Biolegend, San Diego, CA).

After MSCs were treated with 3 mM NMDA (Sigma-Aldrich) or 50 μM MK801 (Sigma-Aldrich) for 24 h, APC anti-mouse C-X-C chemokine receptor type 4 (CXCR4; Biolegend) was used for surface staining to study the effect of NMDA receptor activation on CXCR4 expression in MSCs.

MSCs were harvested and then blocked with Fc receptor blocking agent (BioLegend) for 10 min on ice. The antibodies were added and incubated for 30 min at 4°C in dark. The cells were then washed with 0.1% BSA and fixed with 1% paraformaldehyde in PBS. Cell surface staining was analyzed using a FACSCanto II (Becton Dickinson, Franklin Lakes, NJ) within 24 h of staining. FlowJo software version 7.6.1 (FlowJo, Ashland, OR) was used for data analyses.

#### Cell viability assay.

The Cell Counting Kit-8 (CCK-8; Beyotime Institute of Biotechnology, Shanghai, China), which measures cell viability, is based on the conversion of an orange-colored product from water-soluble tetrazolium salt (WST-8) by dehydrogenases in live cells. In brief, 2 × 10^3^ cells in 100 μl DMEM/F-12 supplemented with 10% FBS were seeded into flat-bottomed 96-well culture plates to evaluate the effects of BLM (0, 0.01, 0.1, 1, 10, and 100 μg/ml) or NMDA (0, 0.1, 0.3, 1, 3, and 10 mmol/l) on MSCs proliferation. When the cell confluence reached 30–40% for the proliferation test, the growth media were supplemented with indicated concentrations of BLM or NMDA for 24 h. The cell numbers were then counted using the CCK-8 assay, and the absorbance of resulting formazan was measured at 450 nm using a microplate reader (Thermo Fisher Scientific) according to manufacturer’s instructions.

#### Cell cycle measurement.

MSCs were treated with 3 mM NMDA for 24 h before replacement of medium to analyze cell cycle profiles. At the time points indicated below, MSC were harvested and fixed in ice-cold 70% ethanol. The cells were then centrifuged and incubated with 50 μg/ml propidium iodide and 100 μg/ml RNase A solution (Beyotime). Cell cycle analyses were performed on a FACSCanto II system (Becton Dickinson). Cell cycle profiles were established using FlowJo 7.6.1 software (FlowJo LLC).

#### Experimental animals and treatment.

Female C57BL/6 mice, aged 8–10 wk and weighing ~20 g, were purchased from JingDa Laboratory Animal (Changsha, China). The mice were intratracheally administered with BLM (Nippon Kayaku, Tokyo, Japan) at 5 mg/kg body wt dissolved in 50 μl normal saline (NS) to induce PF. Control mice received 50 μl of NS instead. The GFP-labeled BM-MSCs (1 × 10^6^ in 100 μl PBS) were transplanted via caudal vein 6–8 h after BLM injection, and PBS was injected as the negative control. The mice were randomly divided into four groups (*n* = 10 for each group) *1*) control: intratracheal injection of NS and tail vein injection of PBS; *2*) BLM: intratracheal injection of BLM and tail vein injection of PBS; *3*) BLM + MSCs: intratracheal injection of BLM and tail vein injection of GFP-labeled MSCs; and *4*) BLM + NMDA-MSCs: intratracheal injection of BLM and GFP-labeled MSCs pretreated with 3 mM NMDA for 24 h. The mice were weighed on the day of BLM administration and every day until the end of the experiment. The animals were euthanized at *day 7* or *21* after BLM administration while under anesthesia and exsanguination. The lung tissues were removed and used for the following assays.

This study was approved by the Ethics Committee of Institute of Clinical Pharmacology at Central South University (Changsha, China) and performed in accordance with the guidelines of National Institutes of Health. Before surgeries, the mice were anesthetized with chloral hydrate (400 mg/kg ip), and necessary efforts were taken to minimize suffering.

#### BM cell collection.

After receiving intratracheal administration of BLM, the mice were anesthetized and euthanized at *days 3* or *14* and soaked in 75% alcohol for 5 min. We separated the femur and tibia and placed them in a 35-mm culture dish containing sterilized PBS. The BM cells were rushed into another petri dish with 3 ml of DMEM/F-12 medium until the marrow cavity became white as observed by inserting a 1 ml syringe from both ends of the femur or tibia. The BM cells were gently blown away with a pipette into a single cell suspension, and 5 ml of red blood cell lysis buffer were added per 1 ml of cell suspension. The mixture was lightly blown and centrifuged at 800 rpm for 5 min. The upper red liquid was then discarded. Serum-free culture medium was added for cell precipitation and centrifuged at 800 rpm for 5 min. The upper liquid was discarded, and 5 ml of complete medium containing 10% FBS, 1% penicillin-streptomycin, and 1% l-glutamine were added. Finally, the cells were moved in a 25-cm^2^ culture bottle for BM cells of one mouse and cultured in a humidified CO_2_ incubator at 37°C. Aseptic operation must be considered for the whole process.

#### Amino acid content assay.

After intratracheal BLM administration, the mice were anesthetized and euthanized at *days 3* or *14*. The primary BM cells were then collected and continuously cultured at 37°C in a humidified atmosphere of 5% CO_2_. The supernatants of BM cells were obtained once every 3 days for three consecutive times. The contents of 15 kinds of amino acids in the supernatants were measured by high performance liquid chromatography (HPLC) on the LC-10DVP system (Shimadzu, Kyoto, Japan). Data analysis was based on the original contents of 15 kinds of amino acids in complete medium, which were not used to culture BM cells but placed in the incubator synchronously. The data are from the values that the measured contents of amino acid in the supernatants subtract the original contents of amino acids in complete medium.

BM-MSCs were plated in six-well plates (1 × 10^6^ per well) and incubated with BLM (1 μg/ml) with/without 1 mmol/l l-serine-*O*-sulfate (l-SOS; Sigma-Aldrich) or shxCT transfection for 24 h. The supernatants were collected for Glu content measurement using the Glutamate Assay Kit (Sigma-Aldrich) according to the manufacturer’s instructions.

To knockdown the expression of xCT protein, we used a unique gene-specific short hairpin RNA construct targeting the region of xCT mRNA. The target sequence was 5′-TGTCACTATTTGGAGCTTT-3′.

#### Intracellular reactive oxygen species measurement.

Intracellular reactive oxygen species (ROS) was determined with the Fluorometric Intracellular Ros Kit purchased from Sigma-Aldrich; 1 × 10^6^ cells were plated in 35 mm uncoated glass bottom dishes and treated with 1 μg/ml BLM for 24 h with or without 1 mM *N*-acetylcysteine (NAC; Beyotime) pretreated for 30 min. After treatment, the cells were further loaded with 10 μM DCFH-DA for 30 min in the dark at 5% CO_2_ and 37°C. The unbounded DCFH-DA in medium was removed by being washed twice with PBS. The cells were trypsinized, and the generation of ROS was quantified by measuring the fluorescence using Varioskan Flash (Thermo Fisher Scientific) with excitation at 488-nm wavelength and emission at 525-nm wavelength. The images of fluorescence were visualized directly using a confocal microscope (Leica).

#### Measurement of Ca^2+^.

Changes in the intracellular Ca^2+^ levels in MSCs treated with NMDA were measured by microfluorescent live imaging using Fluo-3 AM (Beyotime Institute of Biotechnology). Loading of MSCs with the fluorescent dye (5 μM) was performed for 60 min at 37°C in the darkness. After loading, the cells were washed with PBS and then incubated for 30 min in HBSS. The cells were placed in an imaging chamber and allowed to settle for 10 min. A series of images (excitation and emission wavelengths at 488 and 525 nm, respectively) was captured using a confocal microscope (Leica). At least five images were taken before the addition of NMDA and were used to calculate the baseline levels of fluorescence.

#### Coculture MSCs with fibroblasts or AEC experiments.

For Transwell coculture experiments, 2 × 10^5^ MSCs were planted in the upper chambers of 0.4-um pore size Transwells (Corning, Lowell, MA). Afterward, 2 × 10^5^ mouse lung fibroblasts (NIH3T3 cells) or mouse lung epitheliums (MLE-12 cells) were placed in six-well culture plates. MSCs were treated with 3 mM NMDA for 24 h. NIH3T3 cells or MLE-12 cells were treated with or without 10 ng/ml of recombinant TGF-β1 (MyBioSource, San Diego, CA) for 24 h. After the medium was reomved and the cells were washed with PBS, the coculture experiments of MSCs with NIH/3T3 cells or MLE-12 cells were conducted in DMEM/F-12 with 2% FBS, 1% l-glutamine, and 1% solution of penicillin-streptomycin in a humidified CO_2_ incubator at 37°C for 24 h. During coculture, 10 ng/ml recombinant hepatocyte growth factor (HGF) from R&D Systems (Abingdon, UK) were used to treat NIH/3T3 cells or MLE-12 cells.

#### Enzyme-linked immunosorbent assay.

Quantification of mouse TGF-β1 (eBioscience, San Diego, CA), HGF (R&D Systems, Minneapolis, MN), and keratinocyte growth factor (KGF; R&D Systems) levels in lung tissue homogenate or cell supernatant was performed by ELISA kits in accordance with the manufacturers’ instructions.

#### Transwell migration assay.

The migration of MSCs was conducted in a Transwell system. The lower surfaces of the filter in 8.0-μm pore size Transwell inserts with 6.5-mm diameter (Corning) were uniformly coated with gelatin. Transwell inserts in 24-well plates were loaded with 1 × 10^4^ MSCs in 250 μl serum-free DMEM/F-12 in the upper chambers. Indicated concentrations of NMDA and MK801, with/without ATI-2341 or WZ811 (Selleck), were added in the upper chambers to treat MSCs. The complete medium with 10% FBS was supplemented in the lower chambers. The cells were allowed to migrate in a humidified CO_2_ incubator at 37°C for 24 h. After the remaining MSCs on the upper surface of the filter were removed with cotton swabs, the cells that migrated to the lower surface were observed and photographed with a fluorescence microscope (Nikon). The average number of cells that migrated was determined by counting the MSCs with green fluorescence in five fields under a microscope (×100).

#### Hematoxylin-eosin staining.

On *day 21*, the right upper lobes of lung tissues were harvested, fixed, and paraffin embedded, followed by sectioning and hematoxylin-eosin staining. The sections were stained with hematoxylin for 5 min, immersed into 1% HCl-ethanol solution to remove excessive hematoxylin, and counterstained with eosin for 3 min.

#### Masson’s trichrome staining and fibrosis score.

The lung sections were stained with Ponceau (Sigma-Aldrich) and acid fuchsin for 5–10 min, treated with 1% phosphomolybdic acid for 5 min, and then counterstained with aniline blue solution (Sigma-Aldrich). All sections were subsequently dehydrated with ethanol, deparaffinized in xylene, and finally mounted with neutral balsam. For semiquantitative analysis of fibrosis by Ashcroft Score, four different levels separated by 100 μm were analyzed by two blinded independent investigators. Morphological changes in fibrotic lungs were quantified according to the criteria of Ashcroft et al. (4).

#### Hydroxyproline assay.

The collagen content in whole mouse lungs was quantified by measuring hydroxyproline (HYP) content in pulverized left lungs as described previously (68). HYP content was measured using a HYP detection kit (Jiancheng Biotechnology Institute, Nanjing, China) according to the manufacturer's protocol.

#### Quantitative real-time PCR.

Total RNA from cells or lung tissues was isolated with TRIzol reagent (Takara) according the manufacturer’s protocol. Total RNA (1 μg) was reverse transcribed into cDNA using a PrimeScript RT Reagent Kit (Thermo Scientific) with gDNA Eraser following the manufacturer’s instructions. Quantitative real-time PCR (RT-qPCR) was conducted using the SYBR Premix Ex Taq II (Takara) in the real-time PCR detection system (CFX96 Touch; Bio-Rad). The relative expression of mRNA was determined by normalizing the expression of each gene to β-actin gene following the 2^−ΔΔCt^ method. The primers are shown in Table 1.

**Table 1.** Quantitative real-time PCR primer

Gene	Forward	Reverse
xCT	5′-CTTTTTGGAGCCCTGTCCTATG-3′	5′-GGATGTAGCGTCCAAATGCC-3′
NR1	5′-ACTCCCAACGACCACTTCAC-3′	5′-GTAGACGCGCATCATCTCAA-3′
NR2A	5′-AGACCTTAGCAGGCCCTCTC-3′	5′-CTCTTGCTGTCCTCCAGACC-3′
NR2B	5′-CCGCAGCACTATTGAGAACA-3′	5′-ATCCATGTGTAGCCGTAGCC-3′
NR2C	5′-ATCGGGGTCAACAATACCAA-3′	5′-CACAGCAGAACCTCCACTGA-3′
NR2D	5′-TAGTGTCAGTGCGCAGATCC-3′	5′-TCCTGGCAGAAGAAGTGGTT-3′
NR3A	5′-CCCTTCCACCTCCCTGAAAT-3′	5′-CAACTTTGCTCCAAGGCTGT-3′
NR3B	5′-CATTCTCTTCGGACGCACTG-3′	5′-AATGTCTTGTCCCCGACCAT-3′
SDF-1	5′-TGCCCTTCAGATTGTTGCAC-3′	5′-TTTTCCTTTTCTGGGCAGCC-3′
CXCR4	5′-GGAAACTGCTGGCTGAAAAG-3′	5′-CTGCTATCCCCCTGACTGAT-3′
Collagen I	5′-GAGCGGAGAGTACTGGATCG-3′	5′-GCTTCTTTTCCTTGGGGTTC-3′
Collagen III	5′-GCACAGCAGTCCAACGTAGA-3′	5′-TCTCCAAATGGGATCTCTGG-3′
SRY	5′-GGCATTTTACAGCCTGCAGT-3′	5′-TGTGCAGCTCTACTCCAGTC-3′
GFP	5′-AGGACGACGGCAACTACAAG-3′	5′-TTCTGCTTGTCGGCCATGAT-3′
β-Actin	5′-TTCCAGCCTTCCTTCTTG-3′	5′-GGAGCCAGAGCAGTAATC-3′

SDF-1stromal cell-derived factor-1; CXCR4, C-X-C chemokine receptor type 4; GFP, green fluorescence protein.

#### Western blot analysis.

Total protein lysates were extracted using RIPA lysis buffer plus proteinase inhibitor cocktail (Roche Diagnostics, Indianapolis, IN). Whole protein concentration for loading was estimated by Bradford assay. The proteins were separated by SDS-PAGE and electrotransferred to PVDF membranes (Millipore). After blocking with 5% BSA, the membranes were incubated at 4°C overnight with β-actin (Sigma), propulmonary surfactant-associated protein C (SP-C; Abcam), α-SMA (Proteintech), fibronectin (Proteintech), collagen I (Santa Cruz Biotechnology), GFP (Abcam), E-cadherin (Cell Signaling Technology), NR1 (Abcam), and xCT (Abcam) Antibodies. The corresponding peroxidase-conjugated secondary antibody (Sigma) was applied for 2 h at room temperature. Immunoreactive bands were detected with chemiluminescence reagents (Millipore) in the Molecular Imager ChemiDoc XRS System (Bio-Rad). The abundance of targeted protein was analyzed using Labwork image analysis software. All experiments were performed at least in triplicate.

#### Immunofluorescence staining.

The lung tissue sections were deparafnized in a xylene series and rehydrated through a decreasing ethanol series for immunofluorescence staining. The slides were pretreated by microwave in citrate buffer (100 mM, pH 7.0) for 10 min and washed three times with PBS. H_2_O_2_ at 3% was used to eliminate the endogenous peroxidase activity. The slides were washed with PBS and incubated with blocking solution for 30 min at room temperature. They were then incubated overnight at 4°C in anti-collagen I antibody dilution (1:50) from Santa Cruz Biotechnology and anti-fibronectin antibody dilution (1:100) from Proteintech Group. Slides were washed with PBS and incubated with the secondary antibody goat anti-rabbit Cy3 (1:200) from Proteintech Group for 1 h at room temperature in the dark. The nucleus was stained with DAPI. The samples were then washed three times with PBS and then analyzed under a fluorescence microscope (Olympus DP72, Tokyo, Japan).

#### Statistical analysis.

The data are presented as means ± SE. Comparisons between two groups were analyzed with unpaired Student’s *t*-test. Comparisons among multiple groups were analyzed with one-way ANOVA, followed by Student-Newman-Keuls test using GraphPad Prism software (GraphPad Software, San Diego, CA). *P* < 0.05 was statistically significant.

## RESULTS

### 

#### Immunophenotype and differentiation potential of BM-MSCs.

**Fig. 1.**
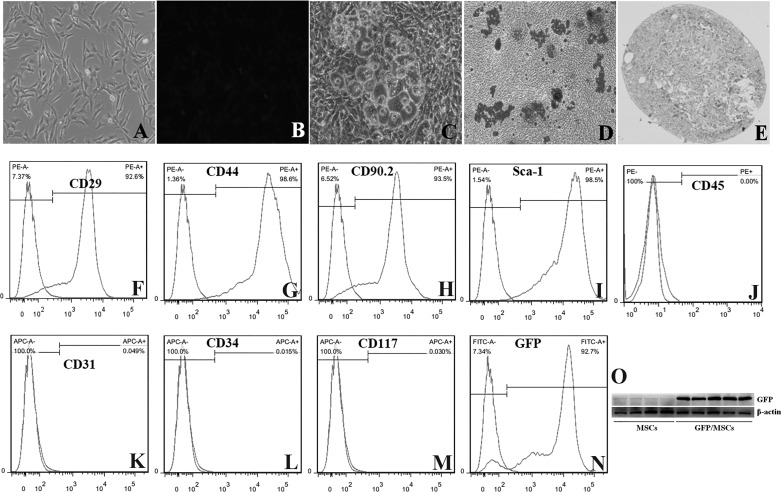
Characteristics of green fluorescence protein (GFP)-labeled bone marrow-derived mesenchymal stem cells (BM-MSCs). The phenotypes of BM-MSCs were confirmed by microscopy and surface markers. BM-MSCs exhibited fibroblast-like phenotype (*A*; ×100) and performed multipotent differentiation potential into adipocytes, osteoblasts, and chondrocytes, as shown by Oil Red O staining (*C*; ×200), Alizarin Red staining (*D*; × 100) and Alcian Blue staining (*E*: ×100). The surface markers were detected by flow cytometry analysis (*F*–*L*). The positive markers were CD29 (*F*), CD44 (*G*), CD90.2 (*H*), and Sca-1 (*I*). The negative markers were CD45 (*J*), CD31 (*K*), CD34 (*L*), and CD117 (*M*). The transfection efficiency of enhanced GFP-lentivirus in BM-MSCs was observed by fluorescence microscopy (*B*; ×100), flow cytometry analysis (*N*), and Western blot assay (*O*).

Considering that MSCs do not have specific marker, enhanced GFP-lentivirus was used to transfect and label BM-MSCs. After transfection, we first examined the delivery of GFP gene in BM-MSCs. The expression of GFP in BM-MSCs was observed by fluorescence microscopy (Fig. 1*B*), Western blot assay (Fig. 1*N*), and flow cytometry analysis (Fig. 1*O*). Detecting surface markers and differentiation potentials is important to confirm the identity of MSCs. Then, the GFP-labeled BM-MSCs were identified by their typical fibroblast-like appearance (Fig. 1*A*) and their differentiation potentials into adipocytes, osteoblasts, and chondrocytes as shown by Oil Red O staining (adipocyte) (Fig. 1*C*), Alizarin Red S staining (osteoblast) (Fig. 1*D*), and Alcian Blue staining (chondrocyte) (Fig. 1*E*). In addition, they were positive for mesenchymal lineage markers, including CD29 (Fig. 1*F*), CD44 (Fig. 1*G*), CD90.2 (Fig. 1*H*), and Sca-1 (Fig. 1*I*) but were negative for hematopoietic lineage markers, including CD45 (Fig. 1*J*), CD31 (Fig. 1*K*), CD34 (Fig. 1*L*), and CD117 (Fig. 1*M*) on the cellular surface by flow cytometry. The BM-MSCs successfully carried the GFP gene and displayed normal immunophenotypic characteristics and differentiation potentials. These results showed the sample cells conformed to the minimal criteria to be identified as MSCs in vitro (14).

#### Glu release was increased from BM cells in BLM-induced PF.

**Fig. 2.**
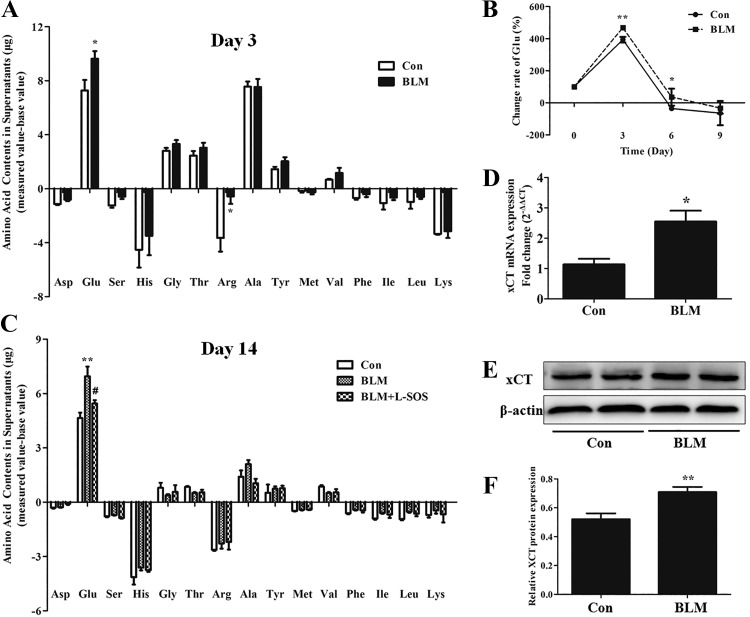
The release of endogenous glutamate (Glu) from bone marrow (BM) cells after bleomycin (BLM)-induced lung injury. *A*: BM cells were isolated from mice at *day 3* after BLM challenge and cultured for 9 days in vitro. The cell supernatants were collected once every 3 days, and 15 kinds of amino acids contents were analyzed by HPLC; *n* = 5–7. *P < 0.05 vs. control (Con). *B*: the change rate of Glu was calculated from the data based on the Glu content in culture medium; *n* = 5–7. *P < 0.05, **P < 0.01 vs. Con. *C*: at *day 14* after BLM challenge, BM cells were separated and treated with 1 mmol/l l-serine-*O*-sulfate (l-SOS) for 3 days in vitro. The cell supernatants were collected for detecting 15 kinds of amino acids contents by HPLC; *n* = 5–7. **P < 0.01 vs. Con; #*P* < 0.01 vs. BLM. *D*–*F*: the total RNA and proteins of BM cells, which were isolated from mice at *day 3* after BLM challenge, were extracted. The mRNA and protein expression levels of xCT were quantified by quantitative RT-PCR and Western blot assay; *n* = 3–5. *P < 0.05, **P < 0.01 vs. Con.

The BM cells of mice were harvested at *day 3* after intratracheal administration with BLM. After the red blood cells were removed, the BM cells were continuously cultured for 9 days in vitro. We collected the supernatants of cultured BM cells once every 3 days for three consecutive times and used HPLC to detect the contents of 15 kinds of amino acids in these supernatants. Data showed that only the content of Glu was higher in BLM group than that in control group (Fig. 2*A*). Using the Glu content of the normal unused medium as a control (*day 0*), we calculated the rate of change of the Glu content in the three collected supernatants. The rate of change in Glu of BLM group was always greater than that of the normal control group, and the content of Glu reached its maximum in the first collected supernatants (*day 3*) (Fig. 2*B*). The process of BLM-induced PF includes the early stage of inflammatory response and the late stage of fibrotic formation (32). The conversion from inflammation to fibrosis occurs around *day 9* after intratracheal instillation of BLM (39). The above results suggested that the release of Glu from BM cells increased in the early inflammatory stage of BLM-induced PF, and the functional status of increased Glu release in BM cells caused by one intratracheal injection of BLM was continued for at least 9 days in vitro.

The BM cells were harvested at *day 14* after BLM challenge and cultured for 3 days in vitro. The supernatants were collected and used to detect the contents of amino acids by HPLC. The results showed that the Glu level was higher in the BLM group than that in the control group (Fig. 2*C*), which revealed the same change trend as that at *day 3* shown in Fig. 2*A*. These results suggested that Glu release from BM cells also increased in the late fibrotic stage of BLM-induced PF, and the functional status of increased Glu release in BM cells caused by one intratracheal injection of bleomycin was continued for at least 14 days in vivo.

The cystine/Glu antiporter (system x_c_^−^) is a major plasma membrane transporter for importing cystine in exchange for Glu and is the critical mechanism for elevating extracellular Glu after stimulation (34, 60). We found that the mRNA and protein expression levels of xCT, the specific catalytic subunit of system x_c_^−^, were significantly elevated in BM cells of BLM-induced PF mice (Fig. 2, *D*–*F*). The BM cells were immediately treated with 1 mmol/l l-SOS, the xCT blocker, after being isolated from the BLM-challenged mice at *day 14* to test the importance of elevated xCT on Glu release during BLM-induced PF. The result revealed that 1 mmol/l l-SOS partially prevented the release of Glu from the BM cells of BLM-induced PF mice (Fig. 2*C*). These data indicated that the system x_c_^−^ played an important role in mediating Glu release from BM cells during BLM-induced PF.

#### BLM caused oxidative stress and Glu release in BM-MSCs via transporter system x_c_^−^ in vitro.

**Fig. 3.**
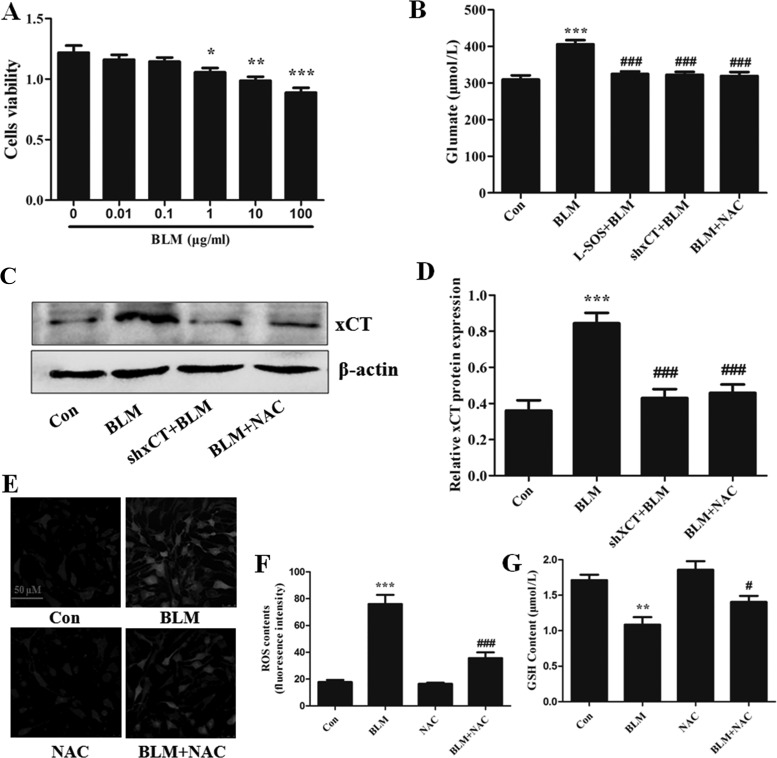
Bleomycin (BLM) caused oxidative stress and increased extracellular glutamate (Glu) in bone marrow-derived mesenchymal stem cells (BM-MSCs) via upregulating xCT. *A*: BM-MSCs were exposed to indicated concentrations of BLM for 24 h. Cell viability was measured by the Cell Counting Kit-8 (CCK-8) proliferation assay; *n* = 3. *P < 0.05, ** *P* < 0.01, ***P < 0.001 vs. 0. *B*: the xCT-specific gene was silenced by shRNA. *B*: the concentration of Glu in the supernatants of cultured BM-MSCs treated with 1 μg/ml BLM for 24 h with/without l-serine-*O*-sulfate (l-SOS; 1 mmol/l), shxCT, and *N*-acetylcysteine (NAC; 1 mmol/l); *n* = 4. ***P < 0.001 vs. control (Con); ###*P* < 0.001 vs. BLM. *C* and *D*: the protein expression level of xCT in BM-MSCs was measured by Western blot assay; *n* = 3. **P < 0.01 vs. Con; #*P* < 0.05 vs. BLM. *E* and *F*: the production of ROS in BM-MSCs treated with 1 μg/ml BLM for 24 h with/without NAC (1 mmol/l). Representative pictures are shown from 3 independent experiments. Bar = 50 μm; *n* = 3. ***P < 0.001 vs. Con; ###*P* < 0.001 vs. BLM. *G*: the synthesis of GSH in BM-MSCs treated with 1 μg/ml BLM for 24 h with/without NAC (1 mmol/l); *n* = 3. **P < 0.01 vs. Con; #*P* < 0.05 vs. BLM.

We further studied whether or not BM-MSCs could release Glu under the stimulation of BLM and tried to validate the role of system x_c_^−^ in mediating the increase in extracellular Glu from BM-MSCs in vitro. BM-MSCs were treated with different concentrations of BLM for 24 h. CCK8 viability assay showed that BLM significantly decreased the cell viability of BM-MSCs at the concentration of more than 1 μg/ml (Fig. 3*A*). Hence, the optimal BLM concentration was 1 μg/ml, which was used in subsequent experiments. We used 1 μg/ml BLM to treat BM-MSCs for 24 h and found that extracellular Glu in the supernatant of cultured BM-MSCs was significantly increased (Fig. 3*B*), and the protein expression of xCT was elevated (Fig. 3, *C* and *D*). The genetic knockdown of xCT with short hairpin RNA in BM-MSCs was performed to inhibit xCT protein expression and further study the role of the system x_c_^−^ in mediating BLM-induced Glu release of BM-MSCs in the in vitro experiment. After l-SOS or shxCT treatment, the effects of BLM-induced increase in Glu release and xCT expression in BM-MSCs disappeared (Fig. 3, *B*–*D*).

The therapeutic application of MSCs was limited due to their susceptibility to oxidative stress, which resulted in the apoptosis of the injected MSCs in injured areas (28). Moreover, the system x_c_^−^ activity was induced by oxidative insults in cultured murine peritoneal macrophages (22). Thus xCT and system x_c_^−^ activities were related to the network of oxidative stress-inducible glutathione (GSH) metabolic enzymes (29). The productions of ROS and GSH in BM-MSCs after stimulation with BLM were analyzed to further observe whether or not Glu release induced by BLM is associated with oxidative stress. We treated BM-MSCs with the ROS scavenger NAC during BLM stimulation. NAC removed the generated ROS and increased GSH content (Fig. 3, *E*–*G*). The increased extracellular Glu and elevated xCT caused by BLM were abolished by NAC (Fig. 3, *B*–*D*). These data revealed that BLM-induced oxidative insults play an important role in mediating system x_c_^−^ activity and Glu release in BM-MSCs.

#### BM-MSCs presented functional expression of NMDAR.

**Fig. 4.**
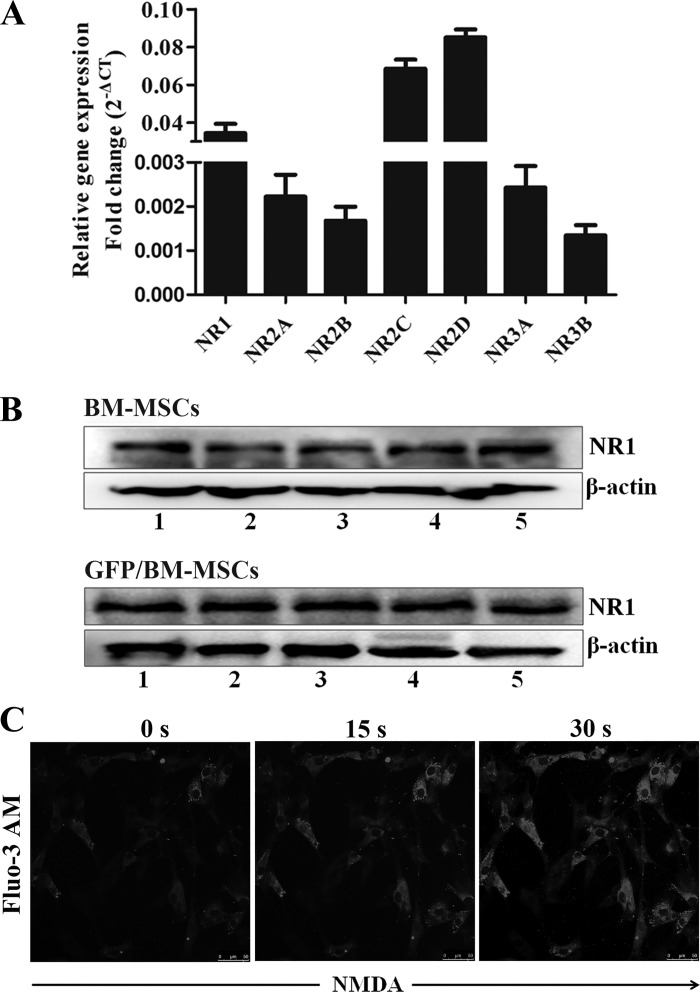
Functional expression of *N*-methyl-d-aspartate (NMDA) receptor (NMDAR) in bone marrow-derived mesenchymal stem cells (BM-MSCs). *A*: the mRNA expression of NMDAR subunits in BM-MSCs was analyzed by quantitative RT-PCR; *n* = 5. *B*: the protein expression of NR1 was quantified by Western blot assay in the primary BM-MSCs (*top*) and the green fluorescence protein (GFP)-labeled BM-MSCs (*bottom*). *C*: the primary BM-MSCs were labeled with Fluo-3 AM and then treated with 3 mM NMDA. Laser confocal took photograph at 488-nm excitation wavelength. Representative pictures are shown from 3 independent experiments.

Glu primarily plays biological roles by acting on its receptor. To explore the effects of Glu release on the functions of BM-MSCs via NMDAR activation, we first examined the expression profile of different NMDAR subunits (NR1, NR2A–D, and NR3A, B) in primary BM-MSCs by RT-qPCR. We found that the seven subunits were expressed in primary BM-MSCs, and the NR2D expression level was the highest (Fig. 4*A*). NR1 is an essential and functional subunit for the formation of a functional NMDAR complex. The protein expression of NR1 was detected in primary BM-MSCs and GFP-labeled BM-MSCs by Western blot assay (Fig. 4*B*). As an ionotropic Glu receptors, NMDAR is a cation-selective ion channel. NMDAR increases the permeability of cell membrane to calcium ion (Ca^2+^) and the influx of extracellular Ca^2+^. We used the Fluo-3 AM assay to observe the Ca^2+^ influx in BM-MSCs after NMDA stimulation by confocal microscope. During NMDA treatment for 30 s, intracellular Ca^2+^ signaling of BM-MSCs was triggered, and fluorescence intensity was continued to enhance (Fig. 4*C*). These results revealed that BM-MSCs had the functional expression of NMDAR and NMDA promoted Ca^2+^ influx in BM-MSCs.

#### NMDAR activation caused BM-MSCs dysfunction in vitro.

**Fig. 5.**
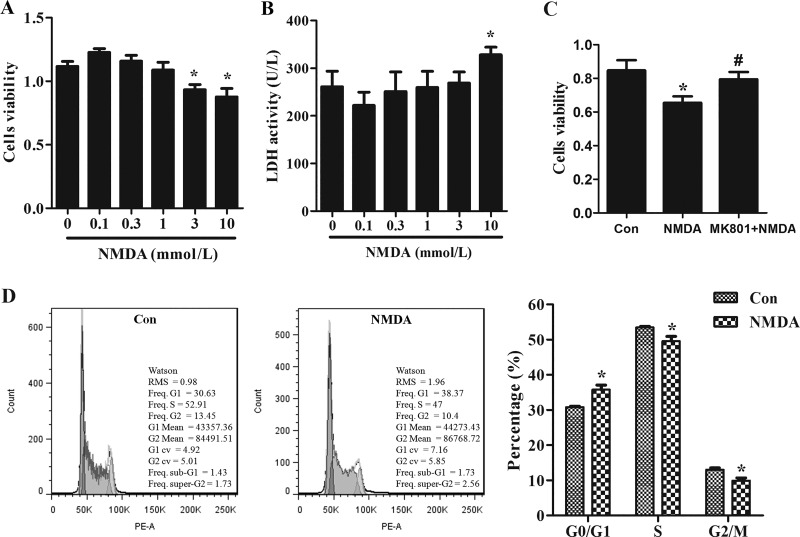
*N*-methyl-d-aspartate (NMDA) receptor (NMDAR) activation inhibited bone marrow-derived mesenchymal stem cell (BM-MSC) proliferation in vitro. *A*: BM-MSCs were exposed to indicated concentrations of NMDA for 24 h. Cell viability was measured by the Cell Counting Kit-8 (CCK-8) proliferation assay; *n* = 3. *P < 0.05 vs. 0. *B*: effect of different concentrations of NMDA treatment for 24 h on the LDH activity in the supernatant of cultured BM-MSCs; *n* = 3. *P < 0.05 vs. 0. *C*: BM-MSCs were treated with 50 μmol/l MK801 for 30 min before exposure to 3 mmol/l NMDA. Cell viability was measured by CCK-8 proliferation assay; *n* = 3. *P < 0.05 vs. control (Con); #*P* < 0.05 vs. NMDA. *D*: cell cycle distribution of BM-MSCs after 24-h exposure to 3 mmol/l NMDA; *n* = 3. *P < 0.05 vs. Con. RMS, root mean squares.

Proliferation, migration, and paracrine functions are important biological characteristics of MSCs. BM-MSCs proliferation was measured by CCK8 assay after being exposed to various concentrations of NMDA. The result revealed that NMDA at the concentration of 3 mmol/l or higher can remarkably inhibit cell proliferation (Fig. 5*A*). LDH activity in the supernatants of cultured BM-MSCs was significantly increased after exposure to 10 mmol/l NMDA (Fig. 5*B*). This finding indicated that 10 mmol/l NMDA can cause cellular injury and destroy the cell membrane. Hence, the optimal NMDA concentration was 3 mmol/l for subsequent experiments. To clarify the decreased proliferation of BM-MSCs after NMDA treatment due to NMDAR activation, BM-MSCs were first treated with the NMDAR antagonist MK801 (50 μmol/l) for 30 min before 3 mmol/l NMDA treatment. MK801 eliminated the decreased viability of BM-MSCs that caused by NMDA (Fig. 5*C*). Cell cycle profiles of BM-MSCs after NMDA treatment were also assessed by flow cytometry. Exposure to 3 mmol/l NMDA for 24 h resulted in more cells in the G_1_ phase and less cells in the S phase and G_2_ phase (Fig. 5*D*). This result revealed that NMDAR activation prolongs cell cycle of BM-MSCs. The migration and paracrine functions of MSCs are crucial for their antifibrotic effects (30). Three millimoles per liter NMDA treatment for 24 h significantly decreased BM-MSC migration to the lower chambers with a high concentration of FBS (see Fig. 9*I*). On the contrary, MK801 (50 μmol/l) weakened the inhibitory effect of NMDA on their migration as compared with the normal control group (see Fig. 9*I*). We subsequently examined the effects of NMDAR activation on the secretion of paracrine factor HGF, a key antifibrotic factor of BM-MSCs (30). NMDA treatment decreased HGF protein content in cultured BM-MSCs supernatants, and this reduction was partially weakened by MK801 (see Fig. 10*A*). In summary, our results demonstrated that NMDAR activation inhibited the proliferation, migration, and HGF secretion of BM-MSCs.

**Fig. 6.**
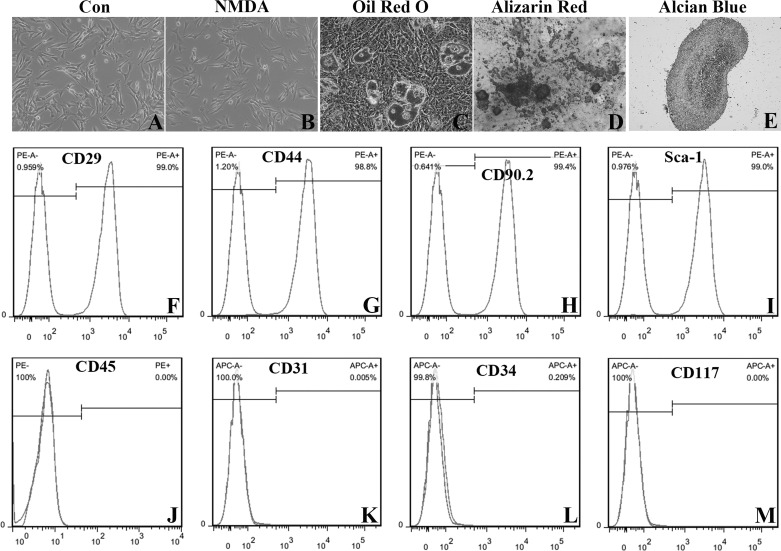
Characteristics of *N*-methyl-d-aspartate (NMDA)-treated bone marrow-derived mesenchymal stem cells (BM-MSCs). BM-MSCs had a spindle fibroblast-like appearance (*A*; ×100). They were treated with 3 mM NMDA for 24 h. Their morphology was normal (*B*; ×100). Trilineage differention, adipogenesis, ostegenesis, and chondrogenesis, were confirmed by Oil Red O staining (*C*; ×400), Alizarin Red staining (*D*; ×400) and Alcian Blue staining (*E*; × 100) respectively. BM-MSCs were positive for CD29 (*F*), CD44 (*G*), CD90.2 (*H*) and Sca-1 (*I*) and negative for CD45 (*J*), CD31 (*K*), CD34 (*L*), and CD117 (*M*) with flow cytometry analyses.

In addition, the phenotype and pluripotency of NMDA-treated BM-MSCs were observed by detecting their surface markers and differentiation potentials. Exposure to 3 mmol/l NMDA for 24 h did not result in changes of the spindle fibroblast-like appearance in BM-MSCs (Fig. 6, *A* and *B*). Their trilineage differention, adipogenesis, ostegenesis, and chondrogenesis, was confirmed by Oil Red O staining (Fig. 6*C*), Alizarin Red staining (Fig. 6*D*), and Alcian Blue staining (Fig. 6*E*) respectively. BM-MSCs were still positive for CD29 (Fig. 6*F*), CD44 (Fig. 6*G*), CD90.2 (Fig. 6*H*), and Sca-1 (Fig. 6*I*) and negative for CD45 (Fig. 6*J*), CD31 (Fig. 6*K*), CD34 (Fig. 6*L*), and CD117 (Fig. 6*M*) with flow cytometry analysis. Thence, these results revealed that NMDAR activation did not change the phenotype and pluripotency of BM-MSCs.

#### NMDAR activation eliminated the beneficial effects of BM-MSCs on BLM-induced PF.

**Fig. 7.**
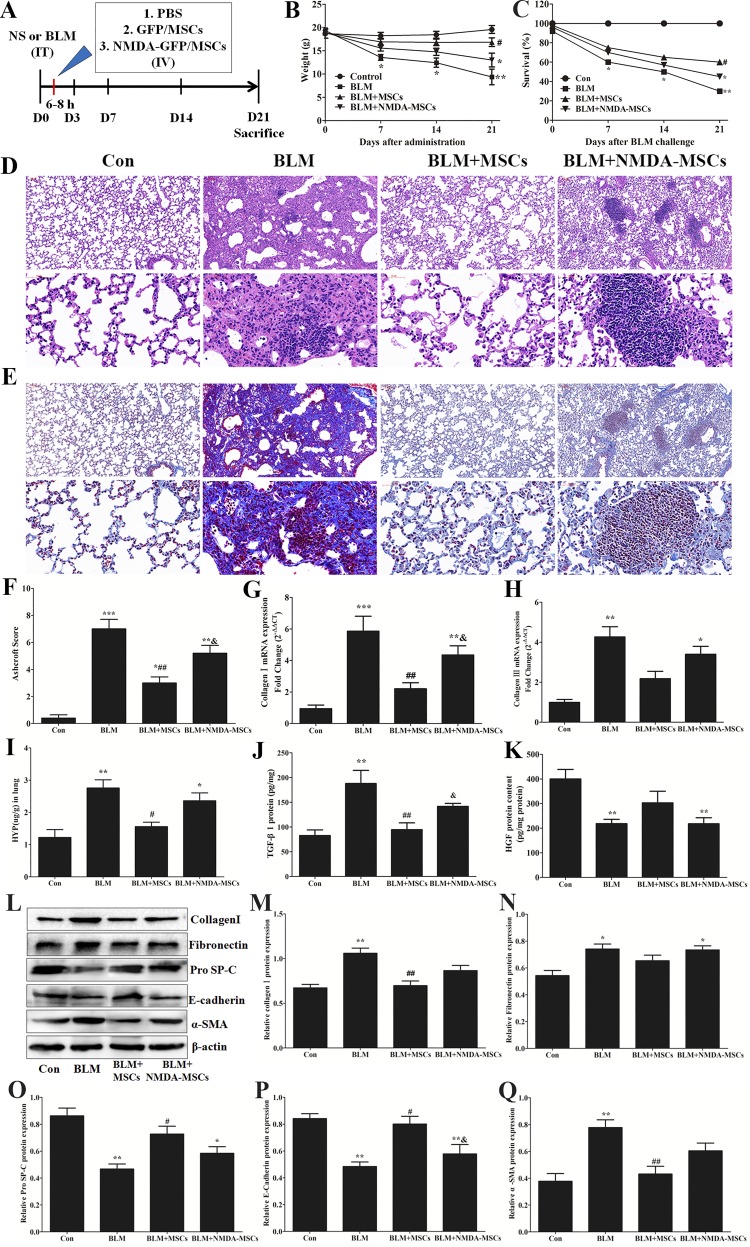
Transplantation of *N*-methyl-d-aspartate (NMDA)-preconditioned bone marrow-derived mesenchymal stem cells (BM-MSCs) aggravated bleomycin (BLM)-induced pulmonary fibrosis. *A*: schematic of experimental design. C57BL/6 mice received BLM by intratracheal instillation, followed by intravenous injection of 1 × 10^6^ green fluorescence protein (GFP)-labeled BM-MSCs or NMDA-preconditioned GFP-labeled BM-MSCs in 100 μl PBS at 6–8 h later, and then euthanized at *days 7* or day *21*. *B* and *C*: weight changes (*B*) and survival analyses (*C*) of mice over a 21-day period after intratracheal BLM administration. *D* and *E*: representative images of hematoxylin-eosin staining (*D*) and Masson’s trichrome staining (*E*) of lung tissues at *day 21* after BLM challenge from different experimental groups. *Top*: magnification: ×100; *bottom*: magnification ×400. *F*: fibrotic changes in lungs were quantified by using Ashcroft scores, ranging from 0 (normal lung) to 8 (complete fibrosis). *G* and *H*: the mRNA expression levels of collagen I (*G*) and collagen III (*H*) in lung tissues at *day 21* were quantified by quantitative RT-PCR. *I*: collagen contents of lung tissues were determined by hydroxyproline assay. *J* and *K*: protein levels of transforming growth factor-β1 (TGF-β1; *J*) and hepatocyte growth factor (HGF; *K*) in total lung homogenates at *day 7* were quantified by ELISA analyses. *L*–*Q*: the protein expression levels of collagen I, fibronectin, prosurfactant protein-C, (SP-C), E-cadherin, and α-smooth muscle actin (α-SMA) in lung tissues at *day 21* were quantified by Western blot analyses; *n* = 3–5. *P < 0.05, **P < 0.01, ***P < 0.001 vs. control (Con); #*P* < 0.05, ##*P* < 0.01 vs. BLM; &*P* < 0.05 vs. BLM + MSCs.

BM-MSC transplantation significantly attenuates BLM-induced lung damage and ameliorates the fibrotic process (45). BM-MSCs were pretreated with 3 mmol/l NMDA for 24 h before tail vein injection to BLM-challenged mice to explore the effect of NMDAR activation on BM-MSC antifibrotic role in BLM-induced PF. The mice were treated with intratracheal BLM or saline (as control), followed by the delivery of PBS (as control) or 1 × 10^6^ normal or NMDA-pretreated BM-MSCs at 6–8 h after BLM challenge (*day 0*). The changes in body weight and survival rate were recorded before the death of the animal. The results showed that the body weight and survival rate of BLM group were significantly lower than those of the control group. Compared with those of the BLM group, the body weight and survival rate were improved by normal BM-MSCs injection but not by NMDA-pretreated BM-MSCs injection (Fig. 7, *B* and *C*). After BLM challenge, the lungs were harvested at *day 21*, analyzed histologically, and assayed for collagen contents.

Histological examination using hematoxylin-eosin and Masson’s trichrome staining of lung tissues and collagen deposition revealed the typical features of BLM-induced fibrotic response in BLM group (Fig. 7, *D* and *E*). Compared with those of the BLM model group, the morphological alterations of lung fibrosis were significantly reduced in the normal BM-MSC treatment group (Fig. 7, *D* and *E*). The fibrotic morphological alterations in the NMDA-pretreated BM-MSCs treatment group were lighter than those in the BLM model group (Fig. 7, *D* and *E*). This indicated that the NMDA-preconditioned BM-MSCs still have protective effects. However, the fibrotic morphological alterations in the NMDA-pretreated BM-MSCs treatment group were severer than those in the normal BM-MSCs treatment group (Fig. 7, *D* and *E*). Hence, the antifibrotic effect of NMDA-preconditioned BM-MSCs is much less than that of normal BM-MSCs. Modified Ashcroft score was performed, in a blinded fashion, to quantify lung fibrosis. We further performed a biochemical quantification of the collagen protein content by hydroxyproline measure. The results showed that Ashcroft scores and hydroxyproline contents were markedly increased in the lung tissues of BLM group as compared with those of the control group and were partially decreased after injection of normal BM-MSCs. However, NMDA pretreatment eliminated the protective effects of BM-MSCs on Ashcroft scores and hydroxyproline contents (Fig. 7, *F* and *I*). In addition, the main ECM components collagen I and collagen III were increased in the NMDA-pretreated BM-MSCs group compared with those in the normal BM-MSCs group (Fig. 7, *G*, *H*, *L*, and *M*). These analyses revealed that BM-MSCs can reduce the pathological changes and collagen deposition of mice induced by BLM, but NMDAR activation on BM-MSCs can weaken these protective effects.

**Fig. 8.**
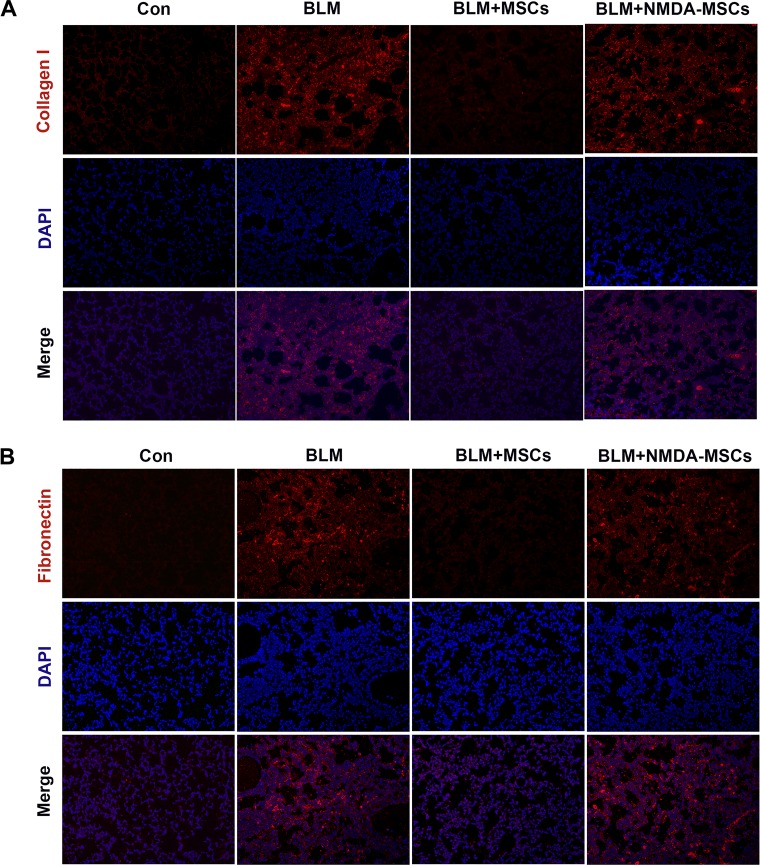
Immunofluorescence staining of collagen I and fibronectin in lungs. Lung slides were stained with anti-collagen I (*A*; ×100) and anti-fibronectin (*B*; ×100) antibodies by fluorescence microscopy. The nucleus was stained with DAPI.

The levels of TGF-β1, which is a key profibrotic factor (67), and HGF, which is an antifibrotic factor and can act as an attractive target for PF treatment (9), in the lung tissues were measured by ELISA assay. After normal BM-MSCs were infused, TGF-β1 was reduced, and HGF was elevated as compared with those in the BLM group (Fig. 7, *J* and *K*). On the contrary, TGF-β1 was upregulated, and HGF was reduced in fibrotic lungs after administration of NMDA-preconditioned BM-MSCs as compared with those in the group administered with normal BM-MSCs (Fig. 7, *J* and *K*). We also evaluated the changes in the molecular expression levels of biomarkers associated with lung injury and fibrosis. The results showed that the expression levels of type II AEC marker (SP-C) and epithelial cell-specific marker (E-cadherin) were markedly decreased, whereas those of interstitial cell-specific markers (α-SMA and fibronectin) were markedly increased in the BLM group compared with those in the normal control group (Fig. 7, *L* and *N–Q*). Therefore, normal BM-MSCs injection restored the expression levels of SP-C, E-cadherin, and HGF and significantly decreased the expression levels of α-SMA, fibronectin, and TGF-β1 as compared with those in the BLM group. However, BM-MSCs didn’t exhibit the above protective effects after NMDA precondition (Fig. 7, *L* and *N–Q*). The protein expression levels of collagen I and fibronectin in lung tissue sections were also measured by immunofluorescence staining (Fig. 8). We got the same results as the above of Western blot results.

In our experiments, BM-MSCs alleviated the lung epithelial cell injury, the accumulation of lung collagen, and the severity of BLM-induced lung fibrosis. However, excessive activation of NMDAR damaged the protective effects of BM-MSCs on BLM-induced PF.

#### NMDAR activation inhibited the homing of BM-MSCs to fibrotic lungs.

**Fig. 9.**
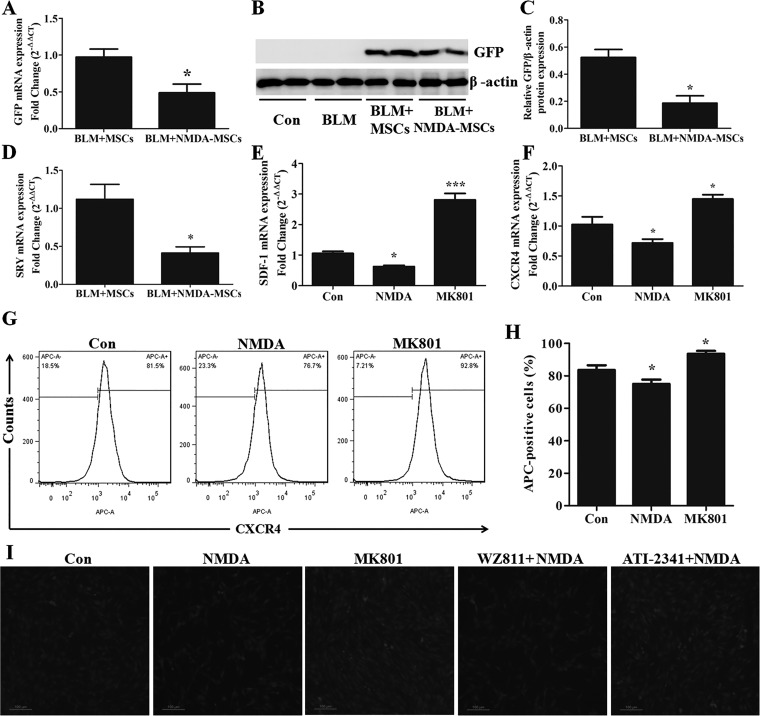
*N*-methyl-d-aspartate (NMDA) precondition inhibited bone marrow-derived mesenchymal stem cells (BM-MSCs) homing and migration via stromal cell-derived factor-1/C-X-C chemokine receptor type 4 (SDF-1/CXCR4) signaling axis. To examine the homing of BM-MSCs in lungs, the mRNA and protein expression levels of green fluorescence protein (GFP; *A*–*C*) and SRY (*D*) genes were detected by quantitative RT-PCR and Western blot assays; *n* = 3–5. *P < 0.05 vs. BLM + MSCs. After being treated with 3 mM NMDA or 50 μM MK801 for 24 h, BM-MSCs were collected and analyzed the mRNA expression levels of SDF-1 (*E*) and CXCR4 (*F*) by quantitative RT-PCR; *n* = 3–5. *P < 0.05, ***P < 0.001 vs. control (Con). *G*: flow cytometry analysis of the percentage of BM-MSCs positive for CXCR4. *H*: the quantitative histogram showed the data of 3 independent BM-MSCs samples of APC-positive cells; *n* = 3. *P < 0.05 vs. Con. *I*: GFP-tagged BM-MSCs were pretreated with 5 μM WZ811 or 1 μM ATI-2341 for 6 h, then treated with 3 mM NMDA or 50 μM MK801 for 24 h. The cell motility of BM-MSCs was determined by Transwell migration assay. Magnification: ×100.

MSCs can home to damaged lung tissues after transplantation, reducing inflammation and fibrosis (48). To determine the effect of NMDA precondition on the homing of BM-MSCs to the fibrotic lungs, the GFP-labeled BM-MSCs from the male C57BL/6 mice were preconditioned with NMDA and injected into female mice through tail vein on the day of BLM administration. The number of these exogenous BM-MSCs was observed by analyzing the SRY and GFP genes in the lungs of female transplant recipients. We found that the SRY and GFP genes were not present in the lungs of the normal control and BLM model groups (Fig. 9, *A*–*D*). The expression levels were lower in the NMDA-preconditioned BM-MSCs group than those in the normal BM-MSCs group (Fig. 9, *A*–*D*). These results revealed that the homing of BM-MSCs to injured lungs was reduced after NMDA precondition.

Next, we studied the effect of NMDAR activation on the migration of BM-MSCs and its mechanism in vitro. Chemokine stromal cell-derived factor-1 (SDF-1, also called CXCL-12) is crucial for the migration of MSCs via interacting with its cognate receptor CXCR4 on the cellular surface (59). MSCs can produce various cytokines and chemokines to regulate their migratory properties (8, 23). We used 3 mmol/l NMDA or 50 μmol/l MK801 to treat BM-MSCs for 24 h and then examined the mRNA and protein expression levels of SDF-1 and CXCR4 by RT-qPCR and flow cytometry analyses. The results showed that NMDA decreased the expression levels of SDF-1 and CXCR4, whereas MK801 increased their expression levels in BM-MSCs (Fig. 9, *E*–*H*). The migration of BM-MSCs was measured by the Transwell migration assay. We investigated the migration of GFP-labeled BM-MSCs by observing the number of cells passing through the basement of Transwell inserts under a fluorescence microscopy. The results showed that NMDA inhibited BM-MSC migration, whereas MK801 promoted BM-MSC migration (Fig. 9*I*). To study the role of SDF-1/CXCR4 axis in the negative regulation of NMDAR activation on BM-MSC migration, we used a CXCR4 agonist or antagonist to incubate cells during NMDA treatment. The results showed that ATI-2341, a CXCR4 agonist, partially reversed the reduced number of BM-MSCs which was caused by NMDA treatment (Fig. 9*I*). However, WZ811, a highly potent competitive antagonist of CXCR4, further decreased the reduced number of BM-MSCs (Fig. 9*I*). These results revealed that NMDAR activation inhibited BM-MSC migration by downregulating the SDF-1/CXCR4 signaling axis.

#### NMDAR activation eliminated the inhibitory effects of BM-MSCs on EMT and fibroblast activation by reducing HGF secretion.

**Fig. 10.**
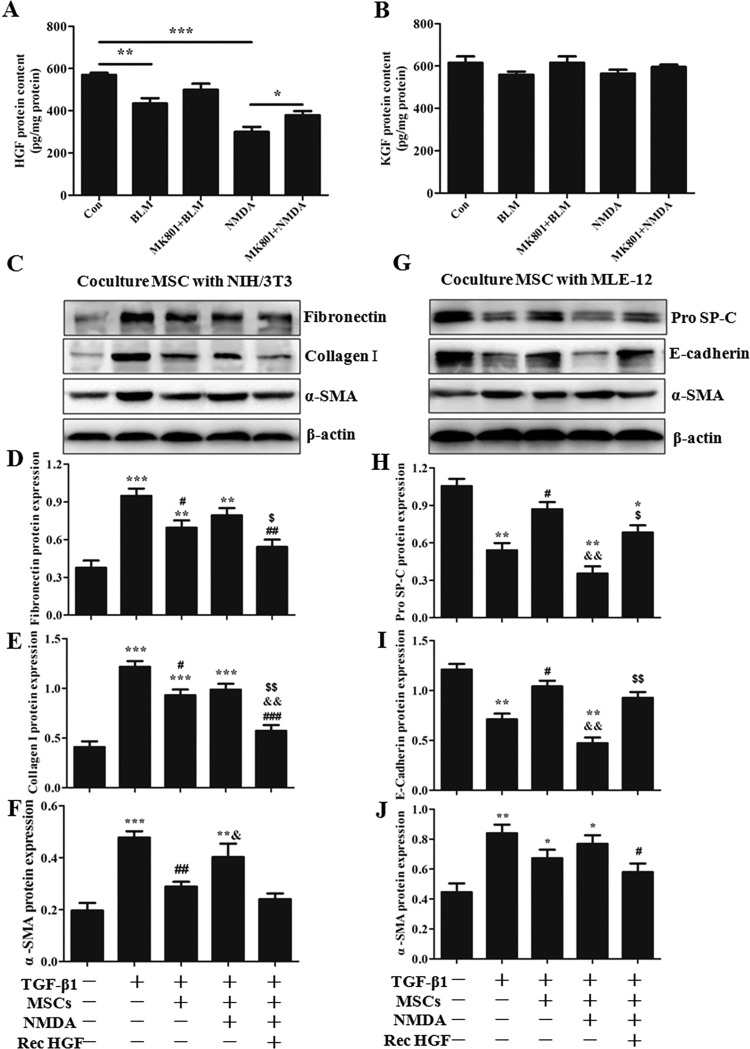
*N*-methyl-d-aspartate (NMDA) receptor precondition eliminated bone marrow-derived mesenchymal stem cells (BM-MSCs) to inhibit fibroblast activation and EMT via reducing hepatocyte growth factor (HGF) secretion. *A*: the secreted HGF in culture media was analyzed by ELISA assay; *n* = 4. *P < 0.05, **P < 0.01, ***P < 0.001. *B*: the secreted keratinocyte growth factor (KGF) in culture media was analyzed by ELISA assay; *n* = 4. To assess the antifibrotic effects of BM-MSCs, normal BM-MSCs or 3 mM NMDA-pretreated BM-MSCs were seeded in the upper chamber, and 10 ng/ml transforming growth factor-β1 (TGF-β1)-treated MLE-12 cells or NIH/3T3 fibroblasts were seeded in the lower chamber in a Transwell coculture system. Simultaneously, MLE-12 cells or NIH/3T3 fibroblasts were treated with 10 ng/ml recombinant HGF. After coculture for 24 h, Western blot assays were performed to determine the protein expression levels of fibronectin, collagen I, and α-smooth muscle actin (α-SMA) in NIH/3T3 cells (*C*–*F*) and the protein expression levels of SP-C, E-cadherin, and α-SMA in MLE-12 cells (*G*–*J*); *n* = 3. *P < 0.05, **P < 0.01, ***P < 0.001 vs. Con. #*P* < 0.05, ##*P* < 0.01, ###*P* < 0.001 vs. TGF-β1; &*P* < 0.05 vs. TGF-β1 + MSCs; $*P* < 0.05, $$*P* < 0.01 vs. TGF-β1+NMDA-MSCs.

In vitro experiments showed that BLM decreased the secretion of HGF protein in the cultured supernatant of BM-MSCs and this decrease can be eliminated by MK801 (Fig. 10*A*). In addition, exogenous NMDA treatment can achieve the same effect of BLM-mediated decrease of HGF secretion (Fig. 10*A*). We also measured another important paracrine factor of MSCs, KGF, which is a crucial epithelial-specific growth factor that plays essential roles in the repair of AECs and the restoration of lung permeability after injury (71). The data showed no difference among the groups after BLM or NMDA stimulation for 24 h (Fig. 10*B*). These results revealed that BLM downregulated the secretion of HGF from BM-MSCs via specifically activating NMDAR but had no effect on KGF secretion.

MSCs can prevent TGF-β1-mediated EMT (15). Fibroblast activation plays a major role in PF development by producing collagens (1). We further studied the role of NMDAR activation of BM-MSCs in TGF-β1-induced EMT and fibroblast activation by the Transwell coculture system. NMDA-preconditioned BM-MSCs were cocultured with TGF-β1-preconditioned NIH/3T3 fibroblasts or MLE-12 cells for 24 h. HGF can inhibit EMT and promote myofibroblast apoptosis (11). We used the recombinant HGF cytokine to treat TGF-β1-preconditioned NIH/3T3 fibroblasts or MLE-12 cells to study the role of HGF secretion from BM-MSCs in EMT and fibroblast activation.

In the coculture system of BM-MSCs with NIH/3T3 fibroblasts, BM-MSCs decreased the protein expression levels of fibronectin, collagen I, and α-SMA in NIH/3T3 fibroblasts as compared with those in the TGF-β1 treatment group. However, the NMDA-preconditioned BM-MSCs increased these protein expression levels (Fig. 10, *C*–*F*). Ten nanograms per milliliter of recombinant HGF partially decreased the protein expression levels of fibronectin, collagen I, and α-SMA in NIH/3T3 fibroblasts (Fig. 10, *C*–*F*).

In the coculture system of BM-MSCs with MLE-12 cells, BM-MSCs decreased the protein expression level of α-SMA and increased the protein expression levels of SP-C and E-cadherin in MLE-12 cells as compared with those in the TGF-β1 treatment group, but these effects were partially abolished by NMDA precondition (Fig. 10, *G*–*J*). However, 10 ng/ml recombinant HGF partially increased SP-C and E-cadherin levels and decreased α-SMA level in MLE-12 cells (Fig. 10, *G*–*J*).

Altogether, above results showed that normal BM-MSCs can inhibit TGF-β1-induced EMT and fibroblast activation, but NMDA preconditioning weakened these effects by specifically reducing HGF secretion.

## DISCUSSION

IPF is a fatal disease that can lead to progressive alveolar structural damage and pulmonary interstitial collagen deposition. Most studies on its pathogenesis have paid attention to local lung fibrosis. However, experimental data revealed that some progenitor cells derived from BM play a crucial role in the fibrogenetic process (41). BM acts as a repository for various stem cell populations, which are mobilized at varying degrees into the peripheral circulation after damage (17). Transplantation of exogenous BM-MSCs, which act as a stem cell therapy, has antifibrosis effects (45). Endogenous BM-MSCs can be mobilized and proceed from the circulatory system to various experimental damaged tissues, where this treatment can promote tissue repair and regeneration by secreting various paracrine factors and directly differentiating into different cells (12). In this study, we first found that BM cells and BM-MSCs released large amounts of endogenous Glu after BLM challenge both in vivo and in vitro. NMDAR activation caused BM-MSC dysfunction in vitro and attenuated their antifibrotic effects on BLM-induced PF in vivo. These findings indicate that BM function is changed, and NMDAR activation in BM-MSCs may be involved in the pathogenesis of BLM-induced PF.

NMDAR functions in erythroid precursor cells and circulating red blood cells and can contribute to intracellular Ca^2+^ homeostasis (36). Studies have indicated that the functional activities of other BM cells, such as neutrophils (6, 42) and platelets (20), are also regulated by NMDAR. Increase in extracellular Glu and subsequent NMDAR activation are induced in various pathological processes (53). In the current research, we first found that BM-MSCs expressed the subunits of NMDAR and NMDAR activation promoted the intracellular concentration of Ca^2+^ in BM-MSCs. These findings indicated that BM-MSCs possessed the functional expression of NMDAR and acted as a NMDAR-regulated target cell. The physiological significance of the functional expression of NMDAR in BM-MSCs is unclear. In addition, when BM-MSCs were exposed to NMDA for 24 h, the long-term activation of NMDAR caused BM-MSC dysfunction, including the inhibition of proliferation, migration, and paracrine in vitro and their homing to fibrotic lungs and antifibrotic effects in vivo.

The cystine/Glu antiporter system x_c_^−^ is an evolutionarily new Glu transport system composed of a light chain, xCT, and a heavy chain, 4F2 heavy chain (4F2hc), which belongs to the family of heterodimeric amino acid transporters (58). 4F2hc is the common subunit of a few other amino acid transporters. xCT is the functional subunit of system x_c_^−^ and exists only in system x_c_^−^. System x_c_^−^ imports cystine in exchange for Glu. By providing the amino acid precursor for antioxidant GSH synthesis, system x_c_^−^ plays a pivotal role in maintaining intracellular GSH levels, extracellular cystine/cysteine redox balance, and extracellular Glu levels (58). In the CNS, system x_c_^−^ activity-mediated Glu release via xCT expression has harmful effects on surrounding neurons (37). In vivo experiments showed that the expression of xCT has been elevated in the CNS, parts of the immune system, and cancer under oxidative stress (34). The enhanced expression levels of xCT were observed in the lungs of paraquat-induced oxidative injury (25) and in LPS-induced endotoxemia mice (62). In the current study, we found that xCT expression of BM cells was elevated in BLM-induced PF mice. In vitro experiments further revealed that BLM caused MSCs to suffer oxidative stress, upregulate the expression of xCT, and promote the release of endogenous Glu. NAC can alleviate the increased xCT expression and Glu release caused by BLM. These results suggested that BLM upregulates the expression of xCT by causing oxidative stress to MSCs and promotes the release of endogenous Glu by upregulating the expression of xCT. In summary, these findings revealed that the release of endogenous Glu via system x_c_^−^-dependent Glu outflow is involved in the pathophysiological process of MSC injury caused by BLM. The mechanisms underlying oxidative stress upregulating the expression of xCT have not been fully elucidated.

Functional NMDARs are heterotetrameric protein complexes composed of two NR1 subunits with NR2 (A–D) subunits or NR3 (A, B) subunits (26). NR2D is the dominant subunit composition of NMDARs, except the functional subunit NR1 in rat lungs, airways, and alveolar macrophages (13). In the current study, we found that the different subunits of NMDAR were expressed in BM-MSCs and NR2D was the dominant subunit. Our previous studies reported that the functional subunit NR1 was expressed in type II AECs and the overactivation of NMDAR downregulated the synthesis of pulmonary surfactants (57). NMDAR activation-mediated proliferation and differentiation of lung fibroblasts promoted the development of hyperoxia-induced chronic lung damage in new born rats (66). However, whether or not large amounts of endogenous Glu released by BM cells will affect the antifibrosis effect of BM-MSCs by activating NMDAR in the process of BLM-induced PF remains unclear. In the current study, BM-MSCs were treated with NMDA in vitro and then transplanted into mice the day after tracheal injection of BLM through tail vein injection to simulate the activated state in vivo. In our experiments, intravenous injection of normal MSCs significantly reduced the BLM-induced lung fibrosis, which is consistent with previous studies. However, the antifibrotic effects of MSCs with NMDAR activated by NMDA in vitro were obviously weakened. The weakened effects include weight loss, decrease in survival rate, lung epithelial cell injury, collagen content deposition, increase in profibrotic gene TGF-β1, and decrease in antifibrotic gene HGF.

To date, intravenous injection of exogenous MSCs as an important stem cell therapy has become a hot topic in PF treatment. Many studies have shown that MSCs have a paracrine function in lung injury repair and regeneration. MSCs possess the capacity to secrete a broad range of bioactive molecules, which can regulate the local immune response to establish a regenerative microenvironment and subsequently inhibit inflammation and promote repair (30). MSC-derived growth factors play essential roles in the repair of pulmonary AECs and vascular endothelial cells and the restoration or maintenance of lung permeability following injury (27). MSCs can also inhibit inflammatory responses by secreting anti-inflammatory mediators (47). In the current study, normal MSC injection significantly decreased the expression levels of fibrosis-related genes (fibronectin, collagen I, α-SMA, and TGF-β1) but increased those of E-cadherin (the epithelium marker) and SP-C (the specific marker of type II AEC). However, these effects of MSCs were abolished by NMDA pretreatment. Therefore, MSCs repaired the injured AECs and downregulated the profibrotic factors in BLM-induced PF mice, but NMDAR activation inhibited the antifibrotic effect of MSCs.

Among the paracrine factors secreted by MSCs, HGF, a pleiotropic growth factor, plays an important role in lung development, inflammation, repair, and regeneration (9, 54). In the animal model of PF, the administration of recombinant HGF protein or ectopic HGF expression ameliorates fibrosis. HGF secreted by MSCs also plays an important role in the protective effect of MSCs against lung injury and fibrosis (7, 18). HGF plays a key role in modulating inflammatory response (9) and wound repair ability (55), in attenuating stimuli-induced EMT and apoptosis in type II AECs (15), and in inhibiting fibroblasts differentiation and ECM production (72). HGF secreted by BM stem cell reduces endoplasmic reticulum stress and improves repair in type II AECs to treat PF (43). In the current study, normal MSC administration increased HGF content of fibrotic lungs induced by BLM, but NMDAR activation inhibited this effect of MSCs in vivo. In vitro experiment showed that BLM and NMDA stimulation inhibited HGF secretion from MSCs through specific NMDAR activation. In the coculture experiments, we found that the effects of NMDAR activation on MSC paracrine function were involved in fibroblast activation and EMT and were exhibited by reducing the secretion of HGF. Therefore, NMDAR activation weakened the beneficial effects of MSCs on lung fibrosis by inhibiting their paracrine function. This phenomenon is an important mechanism explaining that NMDAR activation inhibits the protective effects of MSCs on lung fibrosis.

MSCs play antifibrotic effects also through homing and migrating to injured lung tissues. Our results showed that NMDA suppressed the chemotactic migration ability of MSCs in vitro and reduced the number of MSCs homing to the injured lungs induced by BLM in vivo. This phenomenon may be another important mechanism explaining that NMDAR activation inhibits the ability of MSCs against PF in vivo. SDF-1 is a crucial chemokine for stem cells mobilization by interacting with its cognate receptor CXCR4 on the cellular surface. The expression levels of SDF-1 and CXCR4 were increased in the lungs of patients with IPF as compared with those in normal human lungs. SDF-1/CXCR4 signaling axis mediates the homing and migration of BM-MSCs in BLM-induced lung injury model (69). A significant increase in SDF-1 and CXCR4 mRNA expression was also detected in BM-MSCs of patients with IPF as compared with those of controls (3). In the current study, NMDA decreased the expression levels of SDF-1 and CXCR4. The NMDAR blocker MK801 abolished the decreased expression of SDF-1 and CXCR4 induced by NMDA and restored the chemotaxis activity of MSCs. In addition, the CXCR4 antagonist WZ811 further inhibited the decreased migration ability of MSCs as induced by NMDA, whereas the CXCR4 agonist ATI-2341 abolished the inhibitory effect of NMDA on MSC migration. These results indicated that NMDAR activation inhibited the homing and migration of exogenous MSCs by downregulating SDF-1/CXCR4 signaling pathway.

In conclusion, in the process of BLM-induced PF, the BM cells released a large amount of Glu via the transporter system x_c_^−^. BLM stimulation also increased the Glu release from BM-MSCs via the transporter system x_c_^−^, and NMDAR activation caused BM-MSC dysfunction in vitro. NMDAR activation weakened the antifibrotic effects of BM-MSCs by inhibiting their migration and paracrine effect. This study may provide new insights for further study of the mechanism of PF.

## GRANTS

This work was supported by the National Natural Science Foundation of China Grants 81570065, 81270121, 81100057, and 81370098, Natural Science Foundation of Shanxi Province Grant 201601D01114, Education Department of Hunan Province, Innovation Fund for Institution of Higher Education of Hunan Province Grant 11K076, Fundamental Research Funds for the Central Universities of Central South University Grant 2016zzts115, and Open-End Fund for the Valuable and Precision Instrument of Central South University Grants CSUZC201740, CSUZC201735.

## DISCLOSURES

No conflicts of interest, financial or otherwise, are declared by the authors.

## AUTHOR CONTRIBUTIONS

X.-H.L., C.L., Y.T., Y.-H.H., Q.-M.C., X.-T.H., F.Z., C.-X.H., D.-D.F., J.-P.X., J.H., S.T., W.L., S.Y., and Z.-Q.L. conceived and designed research; X.-H.L. performed experiments; X.-H.L. analyzed data; X.-H.L. interpreted results of experiments; X.-H.L. prepared figures; X.-H.L. drafted manuscript; X.-H.L. and Z.-Q.L. edited and revised manuscript; X.-H.L. and Z.-Q.L. approved final version of manuscript.
